# Advancements and Challenges in Non-Invasive Sensor Technologies for Swallowing Assessment: A Review

**DOI:** 10.3390/bioengineering11050430

**Published:** 2024-04-27

**Authors:** Yuwen Wu, Kai Guo, Yuyi Chu, Zhisen Wang, Hongbo Yang, Juzhong Zhang

**Affiliations:** 1 Suzhou Institute of Biomedical Engineering and Technology, Chinese Academy of Sciences, Suzhou 215163, China; 2 School of Biomedical Engineering (Suzhou), Division of Life Sciences and Medicine, University of Science and Technology of China, Hefei 230026, China

**Keywords:** non-invasive sensors, swallowing assessment, flexible wearable sensors

## Abstract

Dysphagia is a pervasive health issue that impacts diverse demographic groups worldwide, particularly the elderly, stroke survivors, and those suffering from neurological disorders. This condition poses substantial health risks, including malnutrition, respiratory complications, and increased mortality. Additionally, it exacerbates economic burdens by extending hospital stays and escalating healthcare costs. Given that this disorder is frequently underestimated in vulnerable populations, there is an urgent need for enhanced diagnostic and therapeutic strategies. Traditional diagnostic tools such as the videofluoroscopic swallowing study (VFSS) and flexible endoscopic evaluation of swallowing (FEES) require interpretation by clinical experts and may lead to complications. In contrast, non-invasive sensors offer a more comfortable and convenient approach for assessing swallowing function. This review systematically examines recent advancements in non-invasive swallowing function detection devices, focusing on the validation of the device designs and their implementation in clinical practice. Moreover, this review discusses the swallowing process and the associated biomechanics, providing a theoretical foundation for the technologies discussed. It is hoped that this comprehensive overview will facilitate a paradigm shift in swallowing assessments, steering the development of technologies towards more accessible and accurate diagnostic tools, thereby improving patient care and treatment outcomes.

## 1. Introduction

**Figure 1:**
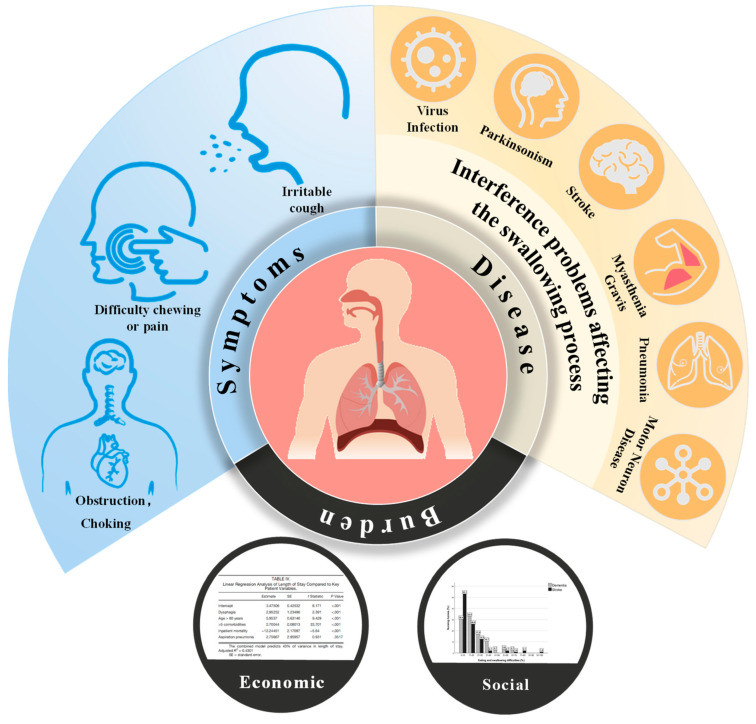
The scope of the impact of dysphagia.

Dysphagia, or difficulty swallowing, presents a significant health challenge affecting a broad spectrum of the population worldwide. It not only diminishes the quality of life for those afflicted but also poses risks of malnutrition and respiratory complications due to the inadvertent inhalation of food or liquids [1]. The complexity of swallowing, requiring coordinated actions of the mouth, pharynx, and esophagus, underscores the intricate nature of its potential dysfunctions [1]. Oropharyngeal dysphagia affects people around the world, with studies showing that the prevalence of dysphagia in the general population varies between 2.3% and 16% [2]. The prevalence of dysphagia varies globally, impacting 2% to 20% of the general population, and is particularly prominent among the elderly, stroke survivors, and individuals with neurological disorders [1]. According to the Agency for Healthcare Research and Quality, 41.7 percent of stroke patients (320,476 patients) in the United States have dysphagia. Considering all causes, the annual number of new cases in the United States reaches 624,757, and approximately 6,288,116 patients currently suffer from dysphagia [3]. Figure 1 shows the scope of the impact of dysphagia.

A study by C. Adkins et al. [1], which surveyed over 31,000 Americans, found that 16.1% of adults were affected by dysphagia. Similarly, Eslick and Talley’s study reported that 16.4% of individuals in Sydney [4], Australia, experienced dysphagia, while an Argentine study identified a 12.9% prevalence of dysphagia among respondents in the past year [5].

Dysphagia is also a geriatric syndrome that affects 10 to 33 percent of older adults [6,7,8,9]. Dysphagia, particularly prevalent among the elderly, stroke patients, and individuals with neurological conditions, poses a significant health concern in nursing homes. Oropharyngeal dysphagia, for instance, affects up to 80% of older adults with Alzheimer’s disease and 60% with Parkinson’s disease, and is found in 37% to 78% of stroke patients [2].

Patients with dysphagia are at a higher risk of developing serious health conditions. Stroke patients with dysphagia, for example, face an increased risk of pneumonia and malnutrition, with a relative risk of 3.17 [10]. Patients with swallowing disorders also have significantly increased lung risk and mortality [11].

Additionally, dysphagia is linked to higher mortality rates and poorer physical performance. In nursing homes, a large cross-sectional study highlighted a six-month mortality rate of 24.7% for residents with dysphagia, compared to 11.9% for those without [10]. Hospitalized patients diagnosed with dysphagia are more likely to succumb to their condition. Clinical symptoms of oropharyngeal dysphagia increase the risk of malnutrition, with national registration data from Norway indicating that 50.5% of nursing home residents screened for dysphagia were at nutritional risk [2].

The aging population contributes to the high prevalence of dysphagia, impacting health economics and healthcare systems significantly. Oropharyngeal dysphagia, associated with multiple complications and a poor prognosis, necessitates a multifaceted treatment approach and is often underestimated in vulnerable or hospitalized patient populations. Dysphagia can extend hospital stays; a study covering 2009 to 2013 in the United States revealed that patients with dysphagia had hospital stays 3.8 days longer and incurred $6243 more in hospital costs than those without dysphagia [7,10]. The study by Allen J et al. also highlighted dysphagia’s impact on increasing hospital stays and costs, particularly for older patients with fractures. Moreover, dysphagia significantly affects patients’ quality of life, impacting their social and mental health. The condition can increase anxiety among patients and caregivers and contribute to caregiver fatigue [11].

A European study found that approximately 30% of respondents avoided eating with others, and 41% felt anxious during meals [12]. The COVID-19 pandemic has further complicated the assessment and management of dysphagia, especially in acute care settings [13]. In Japan, aspiration pneumonia, often resulting from dysphagia, is a leading cause of death among the elderly, underscoring the societal challenge posed by swallowing disorders in aging populations [14,15].

Dysphagia, often a consequence of various medical conditions rather than a disease in itself, significantly impacts patient well-being and healthcare systems. Oropharyngeal dysphagia can arise from stroke, head and neck cancers, and neurological diseases like dementia and Parkinson’s disease, while esophageal dysphagia may be caused by conditions such as esophagitis and achalasia. Both types can result from mechanical issues like tumors or motility problems and may be exacerbated by interventions such as tracheal intubation or medications that affect swallowing reflexes. Aging also contributes to dysphagia, with changes in smell and taste reducing appetite and altering dietary choices, thus affecting swallowing muscles and reducing oral intake efficiency [16]. This can lead to complications like choking and dry mouth, compounded by medications common in elderly care that can further impair swallowing functions. The advancement of dysphagia assessment technologies, including sophisticated diagnostic tools, enhances early detection and intervention, allowing for personalized treatment and better management of the condition. These technologies not only improve diagnostic accuracy and patient outcomes but also reduce healthcare costs by preventing complex treatments and unnecessary hospitalizations. Thus, continuous research and development in this field are essential for enhancing the quality of life for patients and optimizing healthcare resources.

In the current clinical environment, traditional methods for diagnosing dysphagia present several limitations, including the need for interpretation by clinical experts, the potential for complications, and high costs. These challenges have spurred the development of swallowing diagnostic devices towards non-invasive and cost-effective approaches. Non-invasive swallowing diagnostic devices facilitate assessments in a more comfortable and convenient manner, mitigating the discomfort and anxiety associated with traditional examination techniques. Moreover, the adoption of these innovative devices is expected to reduce healthcare costs, decrease reliance on specialized personnel, and enable broader application across various medical settings, including community health centers and home care environments. Thus, by advancing the development of non-invasive and affordable swallowing diagnostic tools, we can enhance the accuracy and accessibility of swallowing function assessments, ultimately improving patient care.

This article aims to provide a reference for the diagnosis and treatment of swallowing disorders by summarizing current methodologies. It begins with an introduction to the physiological mechanisms and stages of swallowing, followed by a detailed description of existing assessment methods and innovations in non-invasive monitoring devices. Lastly, it discusses the challenges and opportunities associated with non-invasive swallowing assessment techniques.

## 2. Methods

### 2.1. Data Collection and Search Strategy

Our literature review encompasses an array of prestigious databases, including Science, IEEE, Springer, Nature, and Google Scholar. The objective was to delineate the progression of swallowing assessment techniques from 2014 onwards. To achieve a more refined search, we utilized the primary keywords “swallowing” and “dysphagia”, supplemented by “evaluation”, “assessment”, and “non-invasive” to narrow down the scope. A meticulous application of inclusion and exclusion criteria facilitated the selection of relevant literature.

### 2.2. Criteria for Literature Inclusion and Exclusion

To ensure a focused and comprehensive evaluation of the methodologies in swallowing assessment, we established specific inclusion and exclusion criteria.

Inclusion Criteria:

- Studies that detail methods of swallowing assessment or introduce devices designed for this purpose.
- Scientific articles that are written in English and accessible for review.

Exclusion Criteria:

- Research focused on devices or methods not directly related to the assessment of swallowing functionality.
- Studies that are limited to evaluating system performance or clinical trials without providing insight into design methodologies.

A structured framework was employed to sift through the extensive volume of literature identified. The goal was to catalog the array of technologies employed in existing devices and to organize them coherently. This organization aims to furnish future researchers and device designers with a comprehensive overview of the field.

The literature was classified into invasive and non-invasive methods, further categorized by the technology of the sensors used. This classification sheds light on the spectrum of methodologies and the technological advancements in the field of swallowing assessment over recent years.

## 3. Results

### 3.1. Anatomy of Swallowing

**Figure 2:**
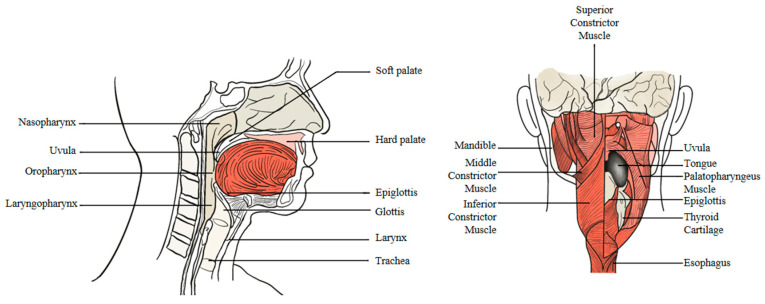
Anatomical diagram of the swallowing site [18].

Understanding the anatomy and physiology involved in the process of eating and swallowing is crucial for diagnosing and treating swallowing disorders, as well as for devising effective rehabilitation plans. The act of swallowing engages over 30 nerves and muscles, showcasing the complexity of this seemingly simple activity [17]. Figure 2 illustrates the anatomical structure of the mouth, pharynx, and larynx, highlighting key components involved in swallowing. The tongue’s musculature, alongside the surface of the mouth and pharynx—demarcated by the tonsillar column—plays a pivotal role. The pharynx houses a layer of constrictor muscles originating from the skull base, hyoid bone, and anterior thyroid cartilage. The submental muscles, emanating from the jawbone, anchor to the hyoid and the tongue, facilitating movement. The cricopharyngeal muscle, vital for the function of the upper esophageal sphincter (UES), originates from the cricoid cartilage. The epiglottis, crucial for preventing aspiration, rises from the throat to attach to the hyoid bone, with the basal lingual fossa situated between it and the pharyngeal surface of the tongue. The larynx, including the true and false vocal cords and the epiglottis, forms the gateway to the lower pharynx, flanked by the pear-shaped fossae on either side [18].

#### Swallowing Muscles

The coordinated action of various muscle groups facilitates the complex process of swallowing, encompassing muscles of the lips and cheeks, jaw, soft palate and hyoid, and the pharynx [18].

- Lips and Cheeks: Involves orbicularis oris, buccinator, risorius, and muscles responsible for elevating and depressing the lips.
- Tongue: Encompasses superior and inferior longitudinal, transverse, vertical, genioglossus, hyoglossus, styloglossus, and palatoglossus muscles.
- Mandibular Muscles: Includes temporal, masseter, lateral and medial pterygoids.
- Soft Palate: Comprises tensor veli palatini, palatoglossus, palatopharyngeus, levator veli palatini, and musculus uvulae.
- Pharyngeal Musculature: Features anterior digastric, geniohyoid, stylohyoid, superior, middle, and inferior constrictors, along with palatopharyngeus and palatoglossus.
- Upper Esophageal Sphincter: Primarily the cricopharyngeus muscle.

The hyoid muscle group, subdivided into superior and inferior hyoid muscles, is integral to the movement of the hyoid bone and larynx during swallowing, ensuring the coordination necessary for the smooth passage of food [19].

### 3.2. Physiology of Swallowing

**Figure 3:**
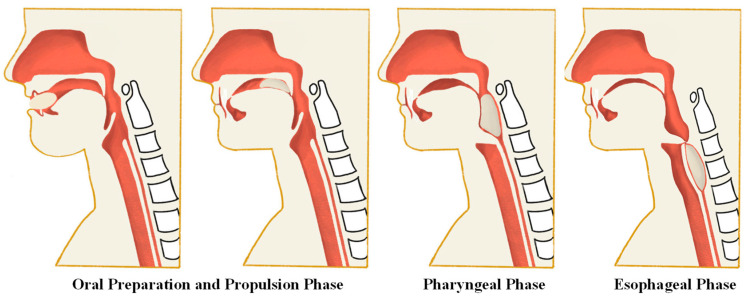
Schematic diagram of the swallowing process.

The swallowing process is traditionally divided into three stages: oral, pharyngeal, and esophageal, each with distinct mechanisms (Figure 3 shows the swallowing process) [18,20,21,22]:

1. Oral Preparation and Propulsion Phase: The oral phase involves the manipulation of food by the tongue, preparation of the bolus with saliva, and its propulsion towards the pharynx. This stage varies in complexity based on the texture of the ingested material, requiring meticulous coordination of sensory feedback and muscle action to prevent premature leakage into the pharynx [18,22].
2. Pharyngeal Phase: This critical phase encompasses a rapid series of events, including pharyngeal peristalsis, UES relaxation, and glottic closure to ensure safe passage of the bolus into the esophagus while protecting the airway from aspiration. The coordinated lifting of the soft palate, retraction of the tongue base, and sequential constriction of pharyngeal muscles facilitate the downward movement of food.
3. Esophageal Phase: The process of food passing through the esophagus involves several steps. Initially, the entry of food prompts peristaltic movements in the esophagus, accompanied by the coordinated opening and closing of the esophageal sphincters, ensuring the smooth transport of the bolus to the stomach. Additionally, the contraction of smooth muscles and the regulation of internal pressure within the esophagus are necessary to facilitate the movement of food.

Understanding these detailed mechanisms of swallowing is essential for identifying dysfunctions and developing targeted treatment strategies, highlighting the intricate balance between anatomical structures and physiological processes.

## 4. Methods of Swallowing Assessment

The literature extensively utilizes measurement methods to assess the presence and extent of oropharyngeal and esophageal swallowing dysfunction. These methods aim to gather objective indicators, including the timing [23,24,25,26,27], pressure [28,29,30], range [31,32,33], and force [34,35] of structural movements, alongside the pattern of bolus flow [36,37,38] and sensory response [39,40] during swallowing.

In vivo measurement, notably, the detection of human physiological responses to oral food handling, has garnered significant interest among food scientists. This approach employs biosensors affixed to the surfaces of human organs and tissues to dynamically monitor physiological responses in a non-invasive manner during the oral handling of food [41,42]. Technically, electromyography and pressure sensing analyses are widely utilized techniques [43]. Furthermore, the acoustic analysis of swallowing sounds, in conjunction with X-ray imaging and ultrasonic pulse Doppler, are also employed to ascertain the speed and timing of bolus passage through the oropharyngeal and hypopharyngeal stages [44,45,46]. Collectively, these studies have laid a robust theoretical foundation for comprehending swallowing abnormalities, thereby advancing the development of diverse assessment methodologies.

### 4.1. Videofluoroscopic Swallowing Study (VFSS)

The VFSS, known as the modified barium swallow (MBS) examination, is widely regarded by swallowing clinicians as the preferred tool and the gold standard for assessing oropharyngeal swallowing [47]. The VFSS offers kinematic analysis, providing detailed visualizations of anatomy and bolus movement, and revealing subtle swallowing abnormalities [48].

This method allows for the detection of the timing and presence of ingested substances at the level of the true vocal cords during swallowing [49,50], aiding in identifying the physiological causes of swallowing disorders. Furthermore, VFSS enables clinicians to assess the impact of various bolus volumes, textures, and compensatory strategies on swallowing physiology [51].

The future trend involves translating VFSS clinical data into quantifiable metrics for diagnosing swallowing disorders. Through frame-by-frame analysis, the VFSS quantifies temporal and kinematic parameters involved in swallowing. Typically, chronological parameters include oral transit time (OTT), soft palate elevation time (SET), hyoid bone movement time (HMT), laryngeal vestibular closure time (LCT), and pharyngeal transit time (PTT). Kinematic parameters include anterior and superior movement of the hyoid bone (HAM, HSM), UES (upper esophageal sphincter) opening, and the pharyngeal constriction ratio (PCR).

However, the VFSS may not always offer the best assessment for every patient and condition. Alternative imaging methods, like flexible endoscopy, can supplement or replace the VFSS in certain cases [47].

The VFSS poses radiation risks, may be difficult to access, and is time-consuming, reliant on clinician expertise, and costly, driving the search for non-invasive, quantitative approaches [52].

### 4.2. Flexible Endoscopic Evaluation of Swallowing (FEES)

FEES stands as a mature auxiliary test for swallowing function beyond the VFSS [53]. It precisely captures the sensory functions of aryepiglottic folds and oropharyngeal sensation to food boluses, playing a crucial role in assessing the protective swallowing reflex and bolus transport in the airway [54,55]. Utilizing a flexible laryngoscope, FEES allows for the observation of the epiglottis, vallecula, tongue base, pharyngeal walls, larynx, and piriform fossae, as well as their movements during various actions. Initially, fiber laryngoscopes were used for these inspections in China. However, with technological advancements, electronic laryngoscopes, offering superior image quality, have largely replaced fiber laryngoscopes, making “flexible laryngoscope swallowing inspection” or “electronic laryngoscope swallowing function evaluation” more appropriate terms today [56,57].

### 4.3. Electromyography (EMG)

**Figure 4:**
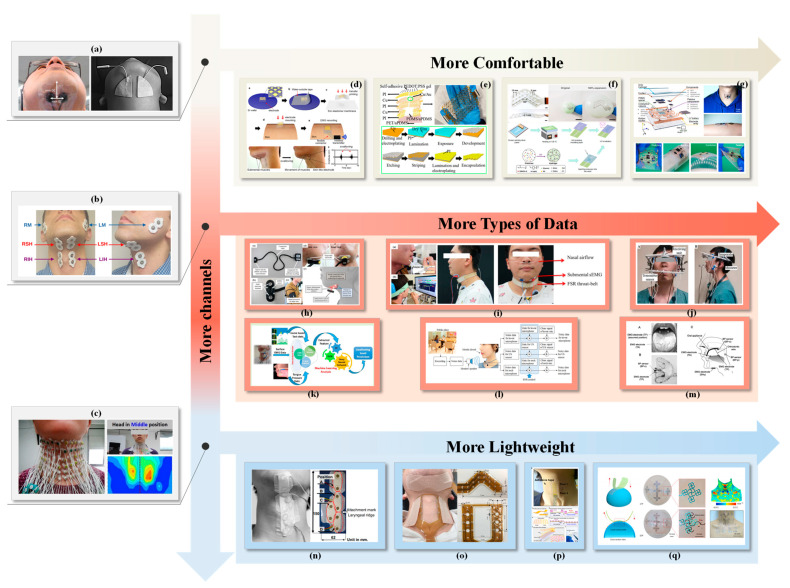
Overview of electromyography in the assessment of swallowing. (**a**) Use of sEMG observation on the jaw muscles swallowing activity; (**b**) Swallowing disorders were analyzed using sEMG and classification models; (**c**) Normal swallowing function was assessed using HD sEMG (96 channels); (**d**) Soft, highly compliant (“skin-like”) electrode; (**e**) A MEMS-based, wearable, flexible embedded high-density sensor; (**f**) A scalable, high-density sEMG electrode array developed by layer-by-layer printing and lamination techniques; (**g**) A fully integrated, self-contained, scalable device; (**h**) A novel muscle function measuring device for simultaneous measurement of submental dMMG and EMG; (**i**) The coordination of respiratory and throat movements is monitored using surface myoelectric sensors, nasal airflow sensors and pressure-sensitive resistance sensors; (**j**) A non-invasive and quantitative swallowing monitoring and evaluation system (including ultrasonic Doppler sensor arrays, microphones, and inertial measurement units); (**k**) An intelligent evaluation system for swallowing based on tongue strength and sEMG; (**l**) An automatic food recognition method combining two modalities of audio and ultrasonic signals; (**m**) The swallowing mechanism was elucidated by means of barometric and EMG measurements; (**n**) To study the factors affecting the measurement of swallowing electromyography; (**o**) The swallowing mechanism was quantified by muscle synergy analysis; (**p**) A tactile sensing multifunctional electronic skin based on patterned metal film; (**q**) Electro-compensation, tattoo-like electrode for epidermal electrophysiology.

Swallowing involves a sequence of voluntary and involuntary muscle contractions. Dysfunction in muscles associated with swallowing can lead to difficulty in performing this critical function [52]. Muscle contraction and relaxation generate weak bioelectrical signals, known as myosignals, that are produced by the electrical activity of neurons within the muscles. These signals can be captured by myoelectric sensors and converted into readable electrical signals through EMG, which plays a significant role in evaluating human activity within man–machine systems. EMG measurements are typically conducted using unipolar or bipolar needle electrodes or surface electrodes [58]. The technique is directly correlated with the degree of muscle contraction and the number of muscles engaged. Dysphagia often results from impaired control by upper motor neurons, and analyzing motor unit morphology offers minimal additional insight; hence, EMG’s primary utility lies in evaluating the relative timing of muscle activity. Table 1 lists recent studies of EMG in the assessment of swallowing. Figure 4 is an overview of electromyography in the assessment of swallowing.

#### 4.3.1. Surface Electromyography (sEMG)

sEMG represents the cumulative effect of superficial muscle EMG and nerve stem electrical activity on the skin’s surface. It generates a one-dimensional voltage time series signal by capturing and recording the bioelectrical changes in the neuromuscular system during both random and non-random activities. sEMG facilitates the indirect, non-invasive analysis of dynamic electrophysiological changes across various swallowing stages. It is predominantly used to assess the electrophysiological impacts of bolus volume and consistency. Although sEMG can be analyzed visually and through amplitude-based measurements, it is subjective, time-intensive, hard to replicate, and prone to amplitude fluctuations [52].

A significant limitation of sEMG is “crosstalk”, where multiple muscles’ activities may interfere with the signal of a targeted electrode, complicating the interpretation of the source signal. This issue is particularly relevant in swallowing sEMG due to the proximity and depth of involved muscles, limiting its application mainly to therapeutic biofeedback [58,81,82].

For effective swallowing evaluation using sEMG, precise electrode placement is essential. The literature suggests that the submental muscle group is the most common placement site, effectively monitoring the submandibular muscle group influencing hyoid bone movement and upper esophageal sphincter opening. Additional placement sites include the masticatory and sublingual muscles.

Recent research efforts have extensively explored sEMG applications. Pathological analyses by researchers such as E. Alfonsi et al. [83], P. Rong et al. [74], and G. Comosentino et al. [84] have shed light on conditions like multiple sclerosis (MS), amyotrophic lateral sclerosis (ALS), and Parkinson’s disease (PD), respectively. 

EMG analyses also contribute to understanding difficulties in consuming various food textures and the relationship between age, gender, and swallowing tasks, revealing a positive correlation between coordination performance and age [15,60,85,86].

A comprehensive understanding of the swallowing mechanism is pivotal for effective dysphagia treatment, and sEMG offers valuable insights by replicating the swallowing process through electrophysiological activities [84,87].

However, sEMG’s broad electrode contact area often fails to isolate specific muscle activities due to adjacent muscle crosstalk. Advances in electrode fabrication technology have led to the development of multi-channel, high-density EMG acquisition systems and flexible electrode sensing technologies. These innovations enable a more detailed analysis of the complex oral and pharyngeal events involved in swallowing [61]. High-density EMG technology addresses traditional surface EMG limitations by providing richer electrophysiological information with higher spatial resolution. This approach has enabled the creation of a more comprehensive reference model for muscle electrophysiological activities and pathological characteristics, laying the groundwork for further research and exploration. High-density sEMG has found applications in diagnosing neuromuscular diseases, assessing muscle conditions, and facilitating human–machine interface control [61,88,89].

Noteworthy contributions include E. Zaretsky et al.’s [60] placement of 42 channels across critical oropharyngeal regions (Device information: The NeoLead electrodes used are pre-wired, made of latex and phthalate/DEHP, and are very compact in size, allowing for high density placement. sEMG signals are recorded using BUCK Elektromedizin’s 16-channel amplifier.) and M. Zhu et al.’s [61] development of a 96-electrode high-density sEMG technique for normal swallowing function assessment (Device information: arranged in a 6 × 16 grid structure with 15 mm spacing between adjacent rows and columns. Each electrode has a round, silver-plated surface with an outer diameter of 10 mm. The HD sEMG signal is filtered using a bandpass filter of 10–500 Hz, and the REFA 128 channel system from TMSi International in the Netherlands records the signal of all channels simultaneously at a sampling rate of 1024 Hz.). However, with the traditional wired connection of electrodes, the cable arrangement is dense and messy, which makes it not easy to operate. Challenges associated with traditional wired electrode connections have prompted innovations such as C. Murakami et al.’s [19] integrated cable arrangement and a flexible 44-channel sEMG sensor for hyoid EMG measurement. The results confirm that the proposed method successfully quantifies the swallowing function from the sEMG signal and maps the signal to the swallowing stage.

Continuing from the advancements in swallowing assessment techniques, further innovations in technology and methodology are crucial for enhancing the understanding and treatment of dysphagia. Among these, the development and optimization of sEMG electrodes have shown significant potential.

The rigid structure of conventional sEMG electrodes often makes it challenging to obtain high-fidelity signals in anatomically complex areas such as the throat. Researchers like D. Zhang et al. [90] have addressed this challenge by creating a scalable, high-density sEMG electrode array utilizing layer-by-layer printing and lamination techniques. In this study, GW-PA-Ag electrodes are incorporated between PU films through scalable layer-by-layer printing and lamination. The patch has excellent electrophysiological properties, including overstretch (1000%), skin matching modulus (10 kPa), and strong long-term signal stability. Compared to conventional Ag/AgCl electrodes, the patch has a lower impedance and lower noise, and when combined with the CNN model, it accurately identifies 11 swallowing activities with an 80% classification accuracy.

Another notable advancement is in overcoming impedance and motion artifacts, particularly for skin areas with significant curvature like the neck. N. Zhao et al. [91] have innovated by designing an embedded high-density sEMG sensor with low modulus, low contact impedance, and minimal motion artifacts, achieving a high signal-to-noise ratio (SNR). This sensor’s biocompatible materials and ergonomic design ensure user comfort and durability, even during extensive usage or in dynamic conditions. The HD-sEMG sensor design comprises three key components: a polyimide film (PI) electrode array frame (PEAF), self-adhesive PEDOT:PSS gel electrodes, and a composite of embedded adhesive poly(dimethylsiloxane) (aPDMS)/PDMS super-elastomers. The PEAF features a serpentine structure with double-layered Cu wiring, optimized for flexibility and toughness with an 8 mm electrode pitch and 0.1 mm Cu wire dimensions. This structure is engineered to interface efficiently with the super-elastomer and gel electrodes, minimizing force application while allowing for slight strain, enhancing the sensor’s mechanical and functional integration. The field has also witnessed significant strides in material science and mechanical design, leading to the emergence of electronic skin (e-skin) or epidermal electronics.

These innovations mimic human somatosensory functions, offering unparalleled skin adaptability and comfort. Y. Wang et al. [92] introduced a large-area, soft, breathable, encapsulated electrode that can be transformed into a filamentous snake-like structure through a cut-and-paste method. This design, inspired by the Katan curve, minimizes electrode strain during application on non-flattenable skin surfaces. Coupled with an electrical compensation strategy, this technology effectively eliminates signal interference, offering a broad range of applications from multichannel electrocardiograms to prosthetic limb control.

Furthermore, the integration of sEMG technology into daily life, such as diet monitoring, presents a novel approach to managing and intervening in unhealthy eating habits [93,94]. An eye-type diet monitoring system, leveraging sEMG, enables seamless daily usage, potentially offering valuable insights for chronic disease diagnosis and prevention. The exploration of multimodal data to assess swallowing function underscores the complexity and multidisciplinary nature of dysphagia management.

The exploration of multimodal data to assess swallowing function underscores the complexity and multidisciplinary nature of dysphagia management. Collaborative efforts by researchers across various specialties have led to the use of surface myoelectric sensors, nasal airflow sensors, and pressure-sensing resistance sensors to understand the coordination of respiratory and laryngeal movements comprehensively [63]. D. Park et al. [48] utilized kinematic analysis, high-resolution emptying manometry (HRM), and EMG to investigate the swallowing sequence. W. Ofusa et al. [67] analyzed the timing of the activity of the swallowing organs during the swallowing stage of the mouth and throat by recording pressure (BP) and tongue muscle activity. This study employed a BP sensor (MPL115A1: Freescale Semiconductor, USA) capable of accurately measuring absolute blood pressure within a confined space (sensor dimensions: 5 × 3 × 2 mm), achieving a high time resolution of 3 milliseconds. For SHy muscle recording, a surface electrode (NM-31, Nippon Optronics Co., Ltd, Tokyo, Japan) was symmetrically positioned on the submental muscle on both sides. The EMG signal underwent amplification through a custom-built amplifier (×1000) and underwent bandpass filtering (30–200 Hz) before being sampled at 1 kHz using a 10-bit analog-to-digital converter integrated within a micro-CPU (H8/3694; Renesas Electronics, Tokyo, Japan). R. Sebastian et al. [78] used sEMG and accelerometer-based neck auscultation (Acc) for non-invasive, multimodal screening of dysphagia. The tri-axial accelerometer MMA7361 (NXP Semiconductors), the NI-DAQ 6215 data acquisition system, and custom LabVIEW software developed by National Instruments were utilized for recording Acc signals. sEMG signals were captured with the Noraxon Ultium^®^ EMG system (Noraxon, Scottsdale, AZ, USA). For each signal type (accelerometer or sEMG) and swallowing task, the researchers constructed distinct feature spaces. Additionally, they investigated early feature fusion by integrating the feature spaces generated from each signal type. The analysis employed four classifiers: support vector machines (SVMs), artificial neural networks (ANNs), XGBoost, and k-nearest neighbors (kNNs). The findings underscore the consistent benefits of signal fusion, which enhance classification performance across all swallowing tasks by leveraging the complementary nature of surface EMG and accelerometer data. Notably, accelerometer signals alone consistently outperformed sEMG signals across all tasks. Some researchers used a metal nano-island decorated with a single layer of graphene and a composite piezoresistive sensor coated with a high polymer (PEDOT:PSS) in combination with machine learning to measure the volume of swallowed liquid based on signals obtained from the skin surface. The device was used simultaneously with conventional sEMG and strain measurement [95]. The sensor was tested on 14 patients with disease-free head and neck cancer to monitor swallowing activity in the head and neck [96]. This study integrated a highly sensitive strain sensor with a machine learning algorithm to differentiate swallowing movements from other types of motion and estimate the amount of swallowed water. The estimation of swallowed water volume involved three main steps: signal processing, feature extraction, and algorithm development. Initially, noise was filtered out from both sEMG and strain raw data. Subsequently, specific features of swallowing patterns within the strain data were identified to construct a volume estimation algorithm. Lastly, the algorithm was individually trained for each participant using data from 60 swallows per participant, and a 3-fold cross-validation test was conducted.

In the aspect of signal processing, C. Bürgin et al. [97] proposed a breathing activity detection method based on the Kalman filter.

#### 4.3.2. Needle Electrodes

The principle of needle electrodes in swallowing monitoring is to record electrical activity during muscle contraction by inserting slender electrodes directly into muscle tissue. Electromyography, which can examine the morphology of individual motor units in the muscles of the limb, can indicate muscle weakness or a neurological cause of muscle weakness [98]. While this appears to be a safe procedure [99], it is somewhat uncomfortable for the patient, especially if some time is spent manipulating the needle to ensure that it enters the muscle of interest, which may go deep into other overlapping muscles that are not involved in swallowing. In some cases, an anesthetic is used, which may alter the sensory and motor cues normally used for swallowing [100], thus affecting the interpretation of the results.

In addition, the need for partially voluntary muscle contractions can be challenging for swallowing research, as swallowing is often a spontaneous process that is difficult to partially voluntarily control under experimental conditions. Another challenge with needle electrodes is the accuracy of the depth and placement of the insertion, especially in the pharyngeal muscles, because these muscles are deep and covered by other muscles, and it takes some time to manipulate the electrodes to ensure their accurate placement. In addition, some cases may require the use of anesthetics to reduce the patient’s discomfort, but this may affect the sensation of swallowing and the motor cues, thus affecting the interpretation of the results. Therefore, in needle electrode monitoring, several electrodes are usually inserted at the same time, and the insertion location and depth need to be carefully selected to ensure reliable data acquisition.

### 4.4. Pressure

#### High-Resolution Manometry (HRM)

**Figure 5:**
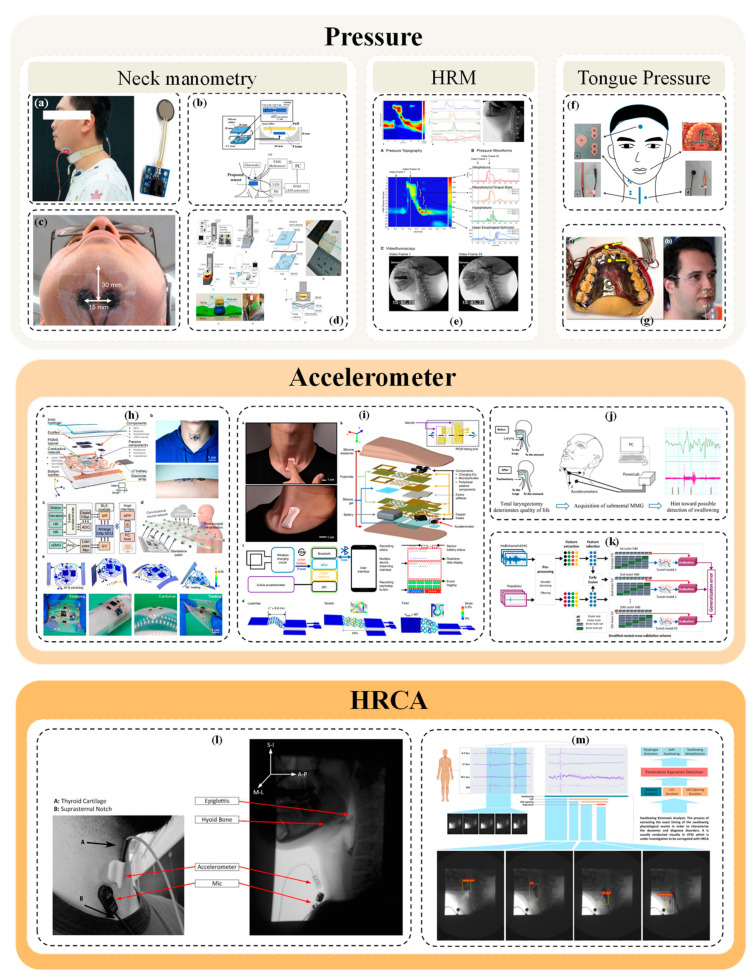
Pressure, accelerometer, and HRCA in the swallowing assessment of relevant studies. (**a**) Coordination of respiratory and throat movements is monitored using surface myoelectric sensors, nasal airflow sensors and pressure-sensitive resistance sensors; (**b**) Development of non-invasive swallowing inspection device with different core optical fiber pressure sensor; (**c**) Neck manometry was used to study the physiological reaction of swallowing gel food and its relationship with texture perception; (**d**) Fiber-optic non-invasive swallowing assessment device based on a wearable pressure sensor; (**e**) HRM combined with radiography was used to simultaneously measure the swallowing pressure at UES and other sites; (**f**) Non-invasive sensing system consisting of pressure sensors, bending sensors, surface electrodes, and microphones; (**g**) Application of a non-personalized light palatometer in the treatment of dysphagia after functional stroke; (**h**) Swallowing features were characterized using MMG; (**i**) Mechanical acoustic sensing of physiological processes and body movements is performed by a soft wireless device (combined with a three-axis accelerometer) placed on the sternal notch; (**j**) To study the differences in anatomical directions of three axis swallowing acceleration measurement signals; (**k**) Study of screening methods for dysphagia using sEMG and accelerometer-based neck auscultation; (**l**) The relationship between hyoid displacement during swallowing and the characteristics of HRCA was analyzed; (**m**) To study the autonomous extraction of neck vibration signal of dysphagia patients based on deep learning.

HRM is an advanced pressure evaluation technique that evolved from traditional hydraulic perfusion manometry [101,102]. It utilizes a catheter equipped with 10 to 36 circumferential or unidirectional pressure sensors spaced 1 cm apart, creating a detailed pressure profile of the esophagus. These pressure data can be visualized on a Clouse plot, featuring color-coded profiles for intuitive interpretation [103,104]. Figure 5 shows a study on the assessment of pressure in swallowing. HRM has become instrumental in providing detailed insights into pressure generation and temporal dynamics from the palatopharynx to the upper esophageal sphincter, areas not measurable by VFSS. Its sensors, spaced 1–2 cm apart and mounted on solid catheters, enhance sensitivity, reliability, and accuracy over traditional methods [48].

Pharyngeal high-resolution manometry (PHRM) offers a focused assessment of oropharyngeal swallowing physiology. It yields quantitative data unavailable from videofluoroscopy (VFS) or fiberoptic endoscopic evaluation of swallowing (FEES), shedding light on the pathophysiology of swallowing disorders. When combined with impedance (PHRIM), PHRM enables the study of how pressure changes influence bolus flow during swallowing. Pressure-flow metrics have led to the development of predictive algorithms and classification models for swallowing efficiency and residue during VFS. PHRM is valuable for quantitatively assessing pharyngeal pressures and the function of the upper esophageal sphincter [101].

Researchers like J. Regan [101] have utilized PHRM to explore how sensory stimulation affects the biomechanics of pharyngoesophageal swallowing in adults with dysphagia. M. Colevas and colleagues have integrated HRM with radiography to simultaneously record pressures across the soft and hard palate, lower pharynx, and UES, analyzing the impact of bolus volume on pharyngeal swallowing dynamics [105].

Moreover, tongue pressure measurement has emerged as a significant method for swallowing evaluation. The process is driven by the coordinated action of oral and pharyngeal muscles, with the tongue playing a pivotal role in propelling the bolus. Measurements of tongue pressure against the hard palate are crucial for understanding the mechanics of bolus propulsion during the oral phase of swallowing [67]. Instruments measuring tongue strength and endurance provide insights into the swallowing function, highlighting the importance of muscular health in effective swallowing [46,106].

In parallel, technological advancements have led to innovative designs in pressure sensing. LanyxUard and Downma [107] have explored ultra-thin, flexible piezoelectric patches that translate throat movements into precise electrical signals, featuring minimal anatomical interference and high strain resolution. The piezoelectric sensor structure is based on a thin film heterostructure composed of an aluminum nitride (AlN) interlayer (120 nanometers), a molybdenum (Mo) bottom electrode (200 nanometers), a piezoelectric aluminum nitride (AlN, 1 micron) layer, and a molybdenum top electrode (Mo, 200 nanometers). The entire manufacturing process employs standard micromanufacturing techniques, including photolithography and sputter deposition. This method ensures precise layer control and structural integrity essential for optimal sensor performance. Based on an aluminum nitride film on a Kapton substrate, these sensors integrate with wireless technology for easy data transmission [108].

M. Maeda et al. [109] introduced a wearable swallowing assessment device using a profile-core fiber-optic pressure sensor, offering high sensitivity, fit, and linearity. The profuse core fiber bending sensor comprises a single-mode transmission fiber with a 9-micron diameter, along with a 1.7 mm length fiber with a diameter of 5 microns, seamlessly integrated via fusion splicing. This device precisely tracks minor pressure fluctuations due to laryngeal movement, representing a leap forward in non-invasive swallowing diagnostics.

The rise of electronic skin (e-skin) mimicking human somatosensory functions heralds a new era in multi-stimuli sensing, including pressure and temperature. Innovations like patterned metal films (PMFs) on flexible substrates have simplified device configurations and enhanced signal processing, promising scalable e-skin production for various applications, including swallowing assessment.

### 4.5. Bioimpedance

Surface bioimpedance is a technique that measures changes in the electrical resistance of tissues or organs in living organisms. The principle is based on the difference in the ability of biological tissues to conduct current and therefore produce different resistance when the current passes through. During swallowing, the movement of the laryngeal pharynx causes changes in the electrical resistance of the biological tissue, so swallowing function can be studied by measuring these changes.

Specifically, surface bioimpedance is measured by placing electrodes on the surface of the skin and then applying a weak alternating current to the inside of the organism. When an electrical current passes through biological tissue, different types of tissue (such as muscle, fat, bone, etc.) will produce different impedances to the electrical current. Muscle tissue generally has a lower resistance, while adipose tissue and bone tissue have a higher resistance. By measuring the change in impedance as an electric current passes through biological tissue, it is possible to infer the properties and movements of the tissue. In swallowing studies, surface bioimpedance can be used to monitor the electrical resistance changes in tissues during laryngeal movement so as to understand the process and characteristics of swallowing. This technique can provide quantitative information about swallowing function, such as swallowing frequency, swallowing force, etc., and compared with some traditional measurement methods, surface bioimpedance is less intrusive and invasive, so it has potential application prospects in clinical practice [97,110,111].

M. Ohashi et al. conducted a study on the identification of swallowing and vocalization events by electromyography, sound, bioimpedance, and high-resolution manometry. Among them, the HRM accuracy is >99%, followed by the sound and bioimpedance waveform is 98%, and then the EMG waveform is 97%, indicating that the bioimpedance has a fairly reliable ability to distinguish between swallowing and non-swallowing events [110].

### 4.6. Barometric Pressure (BP)

Chewing and swallowing occur primarily during nasal breathing. Human studies analyzing chewing and breathing have found an increase in inhaling airflow during chewing. Similarly, oral breathing during chewing interferes with the normal respiratory cycle and reduces the rate of respiration. This means that proper chewing should take place at the same time as nasal breathing [112].

The mouth is composed of hard parts (such as the hard palate, teeth, and jaw) and soft parts (such as the tongue, soft palate, cheek, bottom of the mouth, and gums), and is divided into small spaces such as the interbuccal space, the subhard palate space, the mesopharyngeal space, and the naso-superior pharyngeal space. These structures constantly change their size and/or amount of space as they operate. These morphological changes may affect the air pressure in the mouth, and therefore, pressure measurements may be a tool for assessing tongue function during swallowing [67].

The principle of barometric measurement in swallowing monitoring is to use sensors to measure the changes in air pressure in the mouth and throat during swallowing. When food or liquid enters the mouth, the air pressure inside the mouth changes as the mouth closes and forms a sealed state. As you swallow, the air pressure in the throat changes because the throat communicates with the mouth. By monitoring the changes in air pressure in the mouth and throat, various stages of the swallowing process can be analyzed, including the oral preparation phase and the oral movement phase. During oral preparation, the air pressure inside the mouth will increase with the entry of food, while during oral exercise, the air pressure inside the mouth will decrease with the swallowing action. At the same time, changes in air pressure in the throat also reflect the activity of the throat muscles and how smoothly food passes through the throat. The use of barometry in swallowing monitoring can help physicians assess the coordination and smoothness of swallowing function, as well as detect the presence of swallowing disorders. By analyzing changes in air pressure in the mouth and throat, objective data can be provided to support doctors in developing more effective treatment plans.

### 4.7. Accelerometer

The mechanical dimensions encompass the movement phenomena of various neck and oropharyngeal structures during swallowing, including the hyoid and larynx, or the opening and closing of the laryngeal vestibule and upper esophageal sphincter [78].

Swallowing accelerometers emerge as a promising non-invasive tool for dysphagia assessment, including penetration–aspiration detection, by employing the accelerometer as a sensor during neck auscultation [113].

The accelerometer measures head or neck movement, thereby indirectly monitoring the swallowing action. Swallowing induces specific movement patterns in the head and neck, which the accelerometer detects and records. Utilizing an accelerometer, the acceleration of an object is measured on three axes (X, Y, Z). In swallowing assessments, accelerometers are strategically placed to capture motion changes during swallowing. The device generates specific acceleration patterns upon swallowing, which the accelerometer detects and converts into a digital signal [114]. Figure 5 shows a study on the assessment of accelerometers in swallowing.

Over the past two decades, numerous studies have utilized signals from accelerometers placed on the thyroid cartilage, i.e., accelerometer-based neck auscultation. These studies have applied acceleration signal processing methods for swallow segmentation and detection, inhalation detection, differentiation and characterization of safe and unsafe swallowing, and detailed characterization of physiological events or conditions.

R. Sebastian et al. [78] introduced a non-invasive, multimodal approach to dysphagia screening using surface electromyography (sEMG) and accelerometer-based neck auscultation. Sejdic E. et al. have employed dual-axis accelerometer (ADXL322, Analog Devices, Massachusetts, USA) signals to classify dysphagia patients’ penetration-aspiration versus healthy swallowing [113]. Based on the wavelet packet decomposition of the swallowing accelerometer signal, combined with linear discriminant analysis as a feature dimensionality reduction method and Bayesian classification, the proposed algorithm can distinguish healthy swallowing from inhalation swallowing with over 90% accuracy.

S. Ervin et al. [115] described a wireless device incorporating an electronic skin with a triaxial accelerometer to capture both mechanical acoustic characteristics and precise kinematics of body movements. The device features a flexible printed circuit board (fPCB) constructed with a 25 μm thick polyimide support layer. It incorporates 12 μm thick rolled, annealed copper tracks (AP7164R, DuPont, Wilmington, CA, USA) on both the upper and lower surfaces, each sealed within a 25 μm polyimide insulation layer (FR1510, DuPont). Key electronic subsystems integrated into the device include a three-axis digital accelerometer (BMI160, Bosch, Stuttgart, Germany), a microcontroller equipped with Bluetooth Low Energy protocol for wireless communication (nRF52832, Nordic Semiconductor, Oslo, Norway), and a wireless inductive charging circuit designed to power a 45 mAh lithium polymer battery. This device, comfortably fitting on the upper margin of the sternum, measures mechanical acoustic signals and vital signs during natural daily activities and movements, using frequency domain analysis and machine learning techniques.

H. Xu et al. [116] demonstrated an independent stretchable device platform for wireless larynx measurement, showcasing a modified composite hydrogel with low contact impedance for high-quality, long-term local muscle electrical signal monitoring. To automate the assessment of laryngeal conditions in both new patients and healthy individuals, the research team developed a 2D-class sequential feature extractor (2D-SFE) utilizing convolutional neural networks (CNNs). This system infers pathological states by classifying physiological events. The collected 1D data, including acceleration and sEMG, are initially transformed into 2D vectors resembling image matrices for processing by the CNN-based 2D-SFE, which consists of 62 filtering layers and 2 classification layers. Within the training model, these processing vectors pass through convolution, pooling, and activation layers, effectively reducing the dimensionality of the feature vectors (FVs). The consistency of the softmax functions in the fully connected layer is evaluated to classify the extracted features into specific targets. The machine learning model has demonstrated outstanding performance, achieving an overall prediction accuracy of 98.2% across the 13 states/features analyzed, thereby highlighting the effectiveness of the CNN-based 2D-SFE in multidimensional vector prediction. The integrated three-axis broadband accelerometer measures large body movements and subtle physiological activities/vibrations, providing a foundation for remote disease monitoring and treatment evaluation.

### 4.8. Myotonometer

A myotonometer assesses muscle tone by applying mechanical stimulation and measuring the muscle tissue’s oscillatory response. It evaluates muscle state based on biomechanical properties like elasticity, viscosity, and damping. Mechanical stimulation is applied to the skin and the oscillatory response of muscle tissue is measured, providing insights into muscle tone [117].

### 4.9. Mechanomyography (MMG)

While SEMG offers reliable bioelectrical signals for muscle activity pattern and strength estimation from the body’s surface, MMG, a mechanical counterpart of electromyography, measures muscle contraction-induced lateral shear waves via surface sensors, reflecting mechanical activity [118]. MMG, detectable as vibrations, correlates with muscle strength and provides insights into motor unit recruitment during muscle contraction, potentially aiding in assessing older adults with a high likelihood of anatomical/neurological disorders [118,119].

A. Mialland et al. [119] used mechanical muscle mapping (MMG) to analyze the mandibular region (including the anterior upper neck muscle and the sole of the tongue). The device utilized the ADXL327 analog accelerometer to capture tongue movements during swallowing. This accelerometer features three axes and a sensitivity of 420 mV/g. The bandwidth for the *X* and *Y* axes is 1600 Hz, while for the *Z* axis, it is 550 Hz. Additionally, the swallow signal was acquired through AD Instrument’s single-axis pulse sensor TN1012/ST, which has a bandwidth of 1600 Hz. Shinich et al. [118] developed a device that can simultaneously measure displacement mechanical electromyography (dMMG) and electromyography (EMG) of the sublingual muscles that play a role in swallowing to assess the properties of both signals during tongue lift. The device consisted of two main components: a dMMG/EMG measurement sensor unit and a separate signal processor unit. The sensor unit, which was compact and lightweight at 42 × 17 × 11 mm and 8 g, was affixed to the muscle. It featured EMG electrodes spaced 24 mm apart, with a light reflector positioned centrally for dMMG measurements, maintaining a 3 mm distance from the skin surface. Connected to the sensor unit via a 550 mm communication cable, the signal processor measured 48 × 35 × 16 mm and weighed 25 g.

### 4.10. Cervical Auscultation (Acoustics and Vibration)

Sound analysis is less invasive and easy to implement in clinical practice [120]. The acoustic monitoring and analysis of the swallowing mechanism is a non-invasive and convenient method for the evaluation of swallowing. Early research on swallowing sound analysis focused on the timing of swallowing events. Subsequently, with the application of digital signal processing technology, it was used to detect swallowing disorders, derive the main characteristics of the swallowing sound, and automatically segment the swallowing sound according to its physiological stage. However, the exact source and mechanism of the swallowing sound remains a challenging question. It is well known that the swallowing sound consists of two distinct components: the initial discrete sound (IDS) and the globular passing sound (BTS) [121]. IDS occurs in the pharyngeal stage and is associated with the opening of the upper esophageal sphincter. BTS occurs in the esophageal stage and is a purring sound associated with peristaltic contractions that push pellets into the esophagus. Some swallows may also have final discrete sounds (FDS). FDS is a short click sound at the end of swallowing that is associated with the opening of the airway and is presumed to result from the opening of the airway.

Cervical auscultation involves recording body-produced sounds and vibrations from the larynx during swallowing, offering a non-invasive, convenient swallowing evaluation method. Sound analysis has evolved from focusing on swallowing event timing to detecting swallowing disorders and characterizing swallowing sounds using digital signal processing technology [122].

High resolution cervical auscultation (HRCA), exploring biofeedback possibilities, incorporates non-invasive sensors (contact microphones, three-axis accelerometers) attached to the anterior laryngeal framework, capturing acoustic and vibration signals during swallowing. Advanced signal processing extracts HRCA signal features for machine learning algorithms, correlating with human-rated VF images, providing insights into swallowing physiology. HRCA has potential as a VF diagnostic aid by classifying swallowing safety, tracking hyoid displacement, and annotating temporal swallowing dynamics events, but its functional capability for noninvasively characterizing physiological events targeted by compensatory swallowing actions remains to be further investigated [123]. HRCA signals were simultaneously collected by a triaxial accelerometer (Model ADXL 327, manufactured by Analog Devices, located in Norwood, MA, USA) and a contact microphone. The accelerometer was powered by a 3V supply (Model 1504, manufactured by BK Precision, located in Yorba Linda, CA, USA), which was also connected to the contact microphone. A linear hybrid model was utilized to explore the relationship between HRCA signal signatures and effortless swallowing. Various supervised machine learning classifiers were employed, including SVM, naive Bayes, decision trees, and linear discriminant analysis. These classifiers utilized HRCA signal features to distinguish between types of swallowing. The classifiers processed all HRCA signal features (*n* = 36), statistically significant features (*n* = 9), and features rendered linearly independent by principal component analysis (PCA). A leave-one-out method was implemented to assess the classification accuracy of the classifiers. Among them, the decision tree and linear discriminant analysis classifiers demonstrated the highest performance. Figure 5 shows a study on the assessment of HRCA in swallowing.

Therefore, many research teams have considered using HECA as a screening tool to evaluate the opening and closing of the upper esophageal sphincter, analyze the similarities between thin liquid barium and water during swallowing, study the relationship with hyoid displacement during swallowing, and detect aspiration [124,125,126,127].

### 4.11. Photoelectric Sensor

Usually, most exercises block the tongue movement inside the mouth, and specific oral movement defects may be overlooked. Therefore, visual representations of tongue movements may provide beneficial feedback.

Currently, most proposed feedback techniques for dysphagia treatment either provide additional extra-oral feedback, target the throat stage of the swallowing process, or use contact pressure measurement. The use of photoelectric sensing devices in the mouth holds promise for visualizing dynamic tongue exercises [128]. The optical sensor employed in the design was a commercial integrated proximity sensor (Type SFH7779, Osram, Munich, Germany), measuring 4 mm × 2.1 mm × 1.35 mm with an LED wavelength of 940 nm. Several other integrated sensors were also evaluated; however, the SFH7779 was determined to perform the best in terms of distance measurement accuracy and footprint. Due to its simplicity and the limited size of the corpus, the k-nearest neighbor (kNN) classifier was selected as the baseline classifier. Dynamic time warping (DTW) served as the distance measure to evaluate the similarity between each tested gesture and those within the training corpus. The final results indicated that the cross-validation accuracy reached at least 97.9%. However, the inter-speaker test accuracy fell to 74% and 61%.

Ear-type sensors, due to their easy installation and ability to provide diverse information with minimal effort, have emerged as highly reliable devices for swallowing function assessment. These sensors can measure various parameters, including chewing times, breathing rates, bite force, meal durations, and tongue movements, by simply being inserted into the ear canal. Yoshimoto [129] and colleagues have developed ear-type sensors with extended elastic regions, enhancing the capability to measure changes near the eardrum by allowing deeper insertion into the ear canal. The headphone sensor was designed to resemble the shape of earbuds. Equipped with a built-in optical distance sensor named “QRE1113” (Fairchild Semiconductor International Inc., Sunnyvale, CA, USA), it utilized infrared LEDs and phototransistors to penetrate the inner ear canal by emitting infrared light. The elasticity of the material adjusts to fit individual ear canal shapes, ensuring comfort and adaptability. The connection of the tympanic membrane through the Eustachian tube to the pharynx reflects movements associated with the opening of the Eustachian tube, providing insights into soft palate movement related to swallowing functions.

### 4.12. Ultrasound

**Figure 6:**
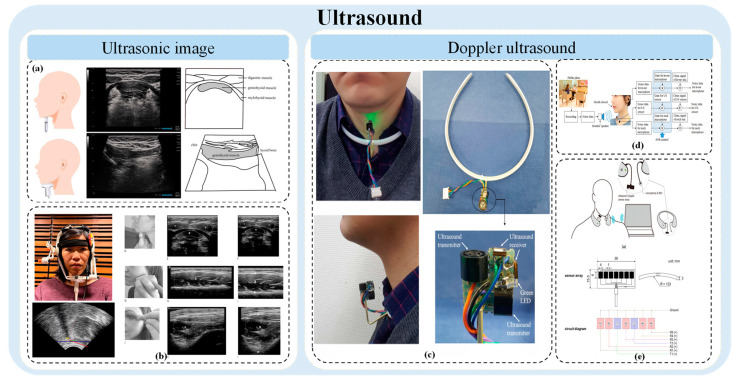
Ultrasound related studies in swallowing assessment. (**a**) The use of ultrasound in the assessment of eating and swallowing; (**b**) Ultrasound was used to automatically assess hyoid movement during normal swallowing; (**c**) Food intake was detected using ultrasonic Doppler sonar; (**d**) Research on automatic food recognition method combining audio and ultrasonic signals; (**e**) A swallowing monitoring and evaluation system based on ultrasonic Doppler sensor was developed.

Ultrasound, a well-established technique for visualizing striated muscles, generates images by detecting reflections caused by tissue density changes. In B-mode imaging, using linear medium to high-frequency probes (5–15 MHz), muscle structures are easily discernable [130]. Striated muscle tissue, for instance, often exhibits a “starry night” appearance in cross-section, showcasing muscle fiber bundles and surrounding septal connective tissue. Longitudinally, muscle fiber arrangements—whether longitudinal, feathery, or triangular—and their angulations are clearly visible. The grayscale value of ultrasound images, indicating the spectrum from black to white, provides crucial diagnostic insights. Healthy muscles typically appear darker, reflecting fewer tissue interfaces between fibers, connective tissues, blood vessels, and nerves. Pathological conditions, by disrupting normal muscle architecture with fibrosis and fat infiltration, render the images whiter. Muscular dystrophies, for example, intersperse muscle with fat and scar tissue, scattering ultrasound waves and producing a “ground glass” appearance. Neurogenic diseases, like motor neuron disease, give a “moth-eaten” appearance, indicating areas of scarring and steatosis adjacent to healthy motor units. In contrast, muscle edema, seen in conditions like dermatomyositis, enhances tissue echo without weakening the ultrasound signal. Ultrasound’s utility has expanded into swallowing function assessment, leveraging the clear correlation between ultrasound image sequences and swallowing functionality. Hyoid advancement, for instance, is investigated as a clinical marker for swallowing evaluation. Figure 6 shows a study on the assessment of ultrasound in swallowing.

By qualitatively analyzing tissue movement and food residue during swallowing, ultrasound provides invaluable insights, especially in assessing oral swallowing disorders and the observation of hyoid movements [131].

#### 4.12.1. Ultrasonic Image

The function of swallowing can be evaluated by ultrasonic observation and qualitative analysis of tissue movement and food residue during swallowing. Ultrasound has been widely used to observe the tongue. Its advantages in the judgment of oral swallowing disorders are recognized by the industry. The evaluation of pharyngeal stage, such as the observation of hyoid movement, has also been explored. However, it is limited by physiological structure and acoustic wave propagation characteristics. In the esophageal stage, there are few studies on ultrasonography. Ma Joan K. et al. [131] used ultrasound imaging to examine hyoid kinematics during swallowing and correlate patterns with different stages of normal swallowing to evaluate the accuracy and robustness of two different automatic hyoid tracking methods compared to manual hyoid position estimation. This study developed two automated trackers intended to potentially enhance the clinical utility of ultrasound in evaluating swallowing. Among these, the DNN (deep neural network) trackers proved to be more robust and accurate compared to shadow trackers. The measurement results obtained from the DNN tracker were highly consistent with those from manual tracking. K. Maeda et al. [132] introduced in detail the process of visualizing and evaluating the structures of mylohyoid muscle, digastric muscle, hyoid muscle, hyoid bone, tongue, masseter muscle, maxillohyoid muscle, orbicularis oris muscle, temporalis muscle, pharynx, esophagus and larynx using ultrasonic images. Based on the extensive experience of ultrasound in neuromuscular diseases, some researchers explained the concept of oral muscle ultrasound and its practical value in the assessment of neuromuscular chewing and swallowing problems [130]. Figure 6 shows a study on the assessment of accelerometer in swallowing.

#### 4.12.2. Doppler Ultrasound

Doppler ultrasound, employing techniques like 40 KHz sine waves, excels in food recognition by detecting specific movement and vibrations based on the type of ingested food [133,134]. This technique, impervious to ambient noise, offers a significant advantage in environments where audio interference is prevalent. The Doppler effect, showcasing frequency shifts relative to movement, aids in recognizing various human activities, including swallowing, by detecting changes in the Doppler frequency due to body part movements. Some studies compared the accuracy of DNN classifiers with methods based on hidden Markov models (HMMs). Given that the food intake process was represented as a time series, recurrent neural networks (RNNs) or long short-term memory (LSTM) networks were employed for food classification. Additionally, the study evaluated a method for determining the emission probability for each HMM class using multi-layer perceptrons (MLPs), referred to as HMM + MLP. In noiseless environments, all DNN-based classifiers outperformed the HMM-based classifiers, with the exception of HMM+MLP. However, in noisy environments, the HMM-based classifiers were more accurate than all DNN-based classifiers [133].

The integration of audio and ultrasonic signals for automatic food recognition demonstrates the potential for comprehensive monitoring systems. Such systems, incorporating ultrasonic Doppler sensors and microphones, specifically focus on swallowing activity, paving the way for non-invasive and quantitative swallowing assessment and monitoring [133].

### 4.13. Other Techniques

Advances in camera imaging methods also show promise for non-contact and remote swallowing evaluation. Techniques like three-dimensional digital image correlation (3D-DIC) and the accelerated segment feature test (FAST) have been explored for detecting muscle wastage in neck muscles and measuring skin displacement during swallowing [135]. The potential of video capture, including smartphone technology, for contactless screening of swallowing disorders underscores the evolving landscape of swallowing assessment tools. These methods, combined with traditional and innovative technologies, offer a holistic approach to understanding and managing swallowing disorders, enhancing diagnostic accuracy and patient care in the realm of biomedical engineering and beyond [136].

Based on the content discussed, the advantages and disadvantages of current mainstream methods for assessing swallowing are outlined in Table 2.

## 5. Prospect and Conclusions

With the development of science and technology, swallowing evaluation technology is facing new opportunities and challenges. Non-invasive sensors provide a more comfortable and convenient way for subjects to be evaluated. By introducing flexible wearable sensors, we see great potential in detecting and treating swallowing dysfunction. The application of patterned metal film, single layer graphene, and other technologies greatly improves the portability and comfort of the sensor and provides a new way for the monitoring of swallowing health.

The research and application of these techniques have brought a new perspective for the detection of swallowing behavior, which not only greatly improves the performance parameters such as sensitivity and anti-fatigue, but also expands the scope of swallowing evaluation from a single sEMG signal to bioimpedance, strain and pressure multi-mode information. Although the perception mechanism of swallowing sensors has been initially established, we still face many challenges. However, despite these advances, challenges remain in terms of universal access, patient compliance, and further improvement in non-invasive techniques to increase sensitivity and specificity.

In future studies, we need to work on addressing these challenges to ensure the effective application and promotion of swallowing assessment techniques.

Of particular concern are swallowing and eating disorders, such as dysphagia, that require long-term monitoring and care. While wearable swallow sensors have shown good stability and reliability in laboratory settings, their ability to monitor over time has not been fully demonstrated in human studies. Future work should therefore focus on long-term evaluation of the sensor’s durability and effectiveness, as well as conducting large-scale human trials to verify its reliability and effectiveness in real-world clinical applications. In addition, future research should focus on improving the accuracy, affordability, and accessibility of dysphagia assessment tools. The development of portable, user-friendly diagnostic devices could democratize dysphagia screening, especially in underserved areas. Artificial intelligence and machine learning can further improve the predictive accuracy of non-invasive assessment methods, enabling personalized treatment regimens. In addition, interdisciplinary collaboration between clinicians, engineers, and data scientists is critical to advance the field of dysphagia research and patient care.

Overall, the development of non-invasive swallowing assessment techniques is promising, but continued research and efforts are still needed. Through unremitting exploration and innovation, we expect to further improve the accuracy and practicality of swallowing assessment technology and provide better diagnoses and treatments for patients with swallowing dysfunction.

## Figures and Tables

**Table 1:** Recent studies of EMG in the assessment of swallowing.

Sensing Modality	Author	Year	EMG Sensor Location	Human Subject	Research Purpose
sEMG	Sebastian Restrepo-Agudelo, Sebastian Roldan-Vasco et al. [59]	2017	Laryngeal girdle muscle area (subcervical)	10 healthy adults	To improve the sEMG detection of infrahyoid muscle during swallowing using digital filtering and discrete wavelet analysis.
sEMG	E. Zaretsky, P. Pluschinski, R. Sader et al. [60]	2017	In the M. masseter, orbicularis oris, submental, and paralaryngeal regions	16 healthy subjects	Identify the most significant electrode locations associated with oropharyngeal swallowing activity.
HD sEMG	Mingxing Zhu, Bin Yu et al. [61]	2017	96 electrodes in the anterior upper neck	12 healthy subjects	A new technique based on high-density surface electromyography (HD sEMG) is proposed for the assessment of normal swallowing function.
Kinematic analysis, HRM, and needle electromyography	Donghwi Park, Hyun Haeng Lee et al. [48]	2017	The superior and inferior hyoid muscles	10 healthy subjects	To investigate the function and importance of the inferior and superior hyoid muscles in the process of swallowing, and to study the swallowing sequence using kinematic analysis, HRM and EMG.
Pressure sensors, bending sensors, sEMG, and microphones	Qiang Li, Yoshitomo Minagi et al. [62]	2017	Maxillary and mandibular muscles	15 adult male subjects without any signs of severe malocclusion	Biomechanical coordination during oropharyngeal swallowing was evaluated based on a non-invasive sensing system.
sEMG, nasal airflow sensor, and pressure sensing resistance sensor	Wann-Yun Shieh, Chin-Man Wang et al. [63]	2019	Mandibular muscle	45 male participants aged 30–50 years. 26 non-smokers and 19 smokers	A study assessing the coordination between swallowing and breathing was carried out using the proposed detection procedure.
SEMG, nasal airflow, and swallowing sounds	Gayathri Krishnan, And S. P. Goswami [64]	2019	The inferior submental muscle of the mandible	30 healthy young volunteers	To study the effects of prone position and gavage volume on swallowing and breathing in healthy young people.
sEMG	Chikako Takeuchi, Eri Takei et al. [65]	2020	Masticatory muscle and sublingual muscle	29 healthy volunteers	To investigate how swallowing behavior is affected by water temperature and water bubble content in healthy people.
sEMG and pressure sensor	Hiroshi Endo, Nobuyuki Ohmori et al. [66]	2020	Mandibular muscle and maxillary muscle	60 healthy volunteers (divided into 2 age groups: young and old)	To investigate the relationship between the temporal characteristics of muscle activity and laryngeal uplift (LE) during swallowing.
Barometric sensor and EMG	Wataru Ofusa, Yoshiaki Yamada et al. [67]	2020	The anterior part of the tongue (TA) and the posterior part of the tongue (TP), as well as the superior pharyngeal constrictor muscle (SHy).	10 healthy volunteers	By recording pressure (BP) and tongue muscle activity, swallowing organs in the mouth and throat swallowing phase of activity time.
sEMG	Johnny McNulty, Kylie de Jager et al. [68]	2021	Submandibular muscles, intercostal muscles, and diaphragmatic muscles	10 participants (5 total laryngectomy (TL), 5 control)	Prediction of laryngeal function by multichannel sEMG classification.
sEMG	JinYoung Ko, Hayoung Kim, Joonyoung Jang, Jun Chang Lee & Ju Seok Ryu [69]	2021	6 channel surface electrodes were placed on the bilateral suprahyoid muscle (SH), bilateral retro-hyoid muscle (RH), thyrohyoid muscle (TH), and thyrosternal muscle (StH)	40 healthy participants (20 older adults older than 60 years and 20 younger adults younger than 60 years)	To study the activation pattern of electromyography during swallowing in the elderly.
sEMG	Sally K. Archer, Christina H. Smith, Di J. Newham [70]	2021	Submentalis	15 people with dysphagia less than 3 months after stroke and 85 healthy participants	Determine whether age or dysphagia after stroke affects increased submental muscle activity during dysphagia, whether sEMG biofeedback improves the performance of dysphagia, and whether the patient receives sEMG as a supplement to treatment.
EMG	Veria Vacchiano, Vitantonio Di Stasi et al. [71]	2021	Masticatory muscle and hyoid muscle	103 people with ALS	To develop a multidimensional facial sEMG analysis for assessing bulbar involvement in amyotrophic lateral sclerosis (ALS).
sEMG	Ben Nicholls, Chee Siang Ang et al. [72]	2022	Masticatory muscles	16 participants	To develop an EMG-based eating behavior monitoring system with haptic feedback to facilitate mindful eating.
sEMG	Mariana M. Bahia, Soren Y. Lowell [73]	2022	M. masseter	20 healthy young adults	To study the sEMG changes in masseter muscle during regular and forced swallowing of saliva.
sEMG	Martin J. McKeown, Dana C. Torpey, Wendy C. Gehm [58]	2022	15 electrodes in the face and throat	7 healthy subjects	A novel approach based on computing independent components (ICs) of simultaneous sEMG recordings to detect potentially functional muscle activation during swallowing using only sEMG electrodes is described.
sEMG	Panying Rong, Gary L. Pattee [74]	2022	The stomatognathic, temporalis, and mandibular abdominis muscles	13 people with ALS and 10 healthy people	To evaluate glossopharyngeal muscle involvement in amyotrophic lateral sclerosis (ALS).
Medical imaging, mandibular kinematics, and EMG	Jianqiao Guo, Junpeng Chen, Jing Wang et al. [63]	2022	Temporalis and masseter	7 healthy volunteers	To establish a subject-specific mandibular modeling framework based on clinical measurements.
sEMG	Wei-Han Chang, Mei-Hui Chen et al. [75]	2023	Anterior temporal muscle, masticatory muscle, and submaxillary muscle	101 subjects with normal swallowing function	Temporal events observed by sEMG were evaluated to elucidate how aging affects coordination between the masticatory and submaxillary muscles.
sEMG	Chiaki Murakami, Makoto Sasaki et al. [19]	2023	Musculus hyoideus	14 healthy young adults and 14 elderly subjects	Based on sEMG through the muscle coordination analysis to quantify the swallowing mechanism.
sEMG (self-made spherical electrodes)	Naoya Saito, Toru Ogawa, Naru Shiraishi, Rie Koide et al. [76]	2023	Masticatory muscles, bilateral abdominal muscles, and hyoid muscles	12 healthy adults	sEMG signals were evaluated to investigate differences in the behavior of masticating and swallowing muscles during spontaneous versus cue swallowing.
sEMG	Sebastian Roldan-Vasco et al. [52]	2023	Masticatory muscle and sublingual muscle group	31 healthy people and 29 people with dysphagia	To study the automatic analysis of sEMG records in healthy people and patients with functional throat dysphagia.
sEMG and ultrasonic image	Ching-Hsuan Pen, Barbara R. Pauloski [77]	2023	Mandibular muscle	24 healthy adults	To explore the effect of real-time ultrasound as visual feedback in MM teaching of healthy adults.
EMG, acoustic, bioimpedance, and high-resolution manometry	Miho Ohashi, Yoichiro Aoyagi, Satoshi Ito et al. [77]	2023	Surface of neck	6 healthy individuals (4 men, 2 women) participated in this study.	Comparison of EMG, acoustic, bioimpedance and high-resolution manometry for identification of swallowing and vocalization events.
sEMG and accelerometer-based neck auscultation (Acc)	Sebastian Roldan-Vasco, Juan Pablo Restrepo-Uribe et al. [78]	2023	Superior and inferior thyroid muscles	30 healthy individuals and 30 patients with functional oropharyngeal dysphagia	A non-invasive, multimodal approach for dysphagia screening using sEMG and accelerometer-based neck auscultation (Acc) was introduced.
EKSS, LPM, pressure, and needle electrode	Enrico Alfonsi, Massimiliano Todisco et al. [79]	2023	Inferior/submental muscle complex	15 healthy subjects	To study the electrodynamics of oropharyngeal swallowing in patients with neurogenic dysphagia.
sEMG and tongue pressure gauge	R. Vaitheeshwari, Shih-Ching Yeh et al. [46]	2023	Laryngeal muscle	8 subjects.	sEMG and tongue pressure gauges were implemented to assess and improve swallowing function in patients with dysphagia.
EMG and sound sensor	Adrien Mialland, Ihab Atallah, Agnès Bonvilain [80]	2024	Hyohyoid muscle and posterior submental muscle, submental muscle	17 participants	Intramuscular electromyography was used to evaluate the hyohyoid and posterior submentalis muscles for feasibility analysis of an implantable active artificial larynx.

**Table 2:** The pros and cons of current mainstream techniques used in evaluating swallowing function.

Method		Advantages	Disadvantages
Videofluoroscopic Swallowing Study (VFSS)		Provides a clear visual representation of food movement from the mouth to the esophagus, identifies issues like pharyngeal residue and aspiration.	Uses X-rays, posing radiation risks; requires special equipment and technical operation, which are costly.
Flexible Endoscopic Evaluation of Swallowing (FEES)		Directly observes the structure and function of the pharynx, can identify disorders such as vagal nerve dysfunction; does not use radiation.	May cause discomfort to patients; the field of view is limited to the scope of the endoscope.
Electromyography (EMG)	Surface Electromyography (sEMG)	Non-invasive, allows for real-time monitoring of muscle activity during swallowing.	Limited to surface muscles, does not provide information about deep muscle activity.
	Needle Electrodes	Can record deep muscle electrical activity in detail, helping to diagnose muscle dysfunction.	Invasive, may cause pain or other complications.
High-resolution manometry (HRM)		Non-invasive, by measuring pressure, it can analyze esophageal swallowing function and pressure changes in detail.	Equipment is expensive and requires professional operation and analysis.
Bioimpedance		Non-invasive, by measuring changes in tissue impedance, it indirectly understands swallowing function.	Data interpretation is complex and can be influenced by various physiological and environmental factors.
Barometric Pressure (BP)		Measures changes in pharyngeal and surrounding air pressure, helping to assess air pressure regulation functions.	Relatively limited technical application, limited data interpretation and practical application.
Myotonometer		Measures muscle stiffness and elasticity, which can assess muscle condition.	May not directly correlate with direct swallowing function.
Mechanomyography (MMG)		Non-invasive assessment of muscle activity through sensing muscle vibrations.	Signals may be disrupted by external noise and movement.
Cervical Auscultation (Acoustics and Vibration)		Non-invasively detects acoustic characteristics during swallowing using sound and vibration sensors.	Interpreting sound data may be subjective and limited in accuracy.
Photoelectric Sensor		Monitors swallowing actions through a photoelectric sensor, simple and non-invasive.	Limited information, difficult to provide in-depth physiological data.
Ultrasound	Ultrasonic Image	No radiation, visually displays tissue structures and movements.	Image resolution and quality are limited by the equipment.
	Doppler Ultrasound	Can accurately identify types of food, have excellent ability to resist environmental noise interference, dynamic monitoring and evaluation can be carried out.	Equipment and operation requirements are high, data interpretation and analysis are complex, and the application range is limited.

## Data Availability

The data used to support the findings of this study are available from the corresponding author upon request.
